# Moving from rhetoric to action: how Africa can use scientific evidence to halt the COVID-19 pandemic

**DOI:** 10.1186/s40249-020-00740-0

**Published:** 2020-10-28

**Authors:** Olushayo Oluseun Olu, Joy Luba Lomole Waya, Sylvester Maleghemi, John Rumunu, David Ameh, Joseph Francis Wamala

**Affiliations:** World Health Organization COVID-19 preparedness and response team, Juba, Republic of South Sudan

**Keywords:** Coronavirus disease 2019, Severe acute respiratory syndrome coronavirus 2, Scientific evidence, Prevention, Control, Africa

## Abstract

The ongoing pandemic of the coronavirus disease 2019 has spread rapidly to all countries of the world. Africa is particularly predisposed to an escalation of the pandemic and its negative impact given its weak economy and health systems. In addition, inadequate access to the social determinants of health such as water and sanitation and socio-cultural attributes may constrain the implementation of critical preventive measures such as hand washing and social distancing on the continent.

Given these facts, the continent needs to focus on targeted and high impact prevention and control strategies and interventions which could break the chain of transmission quickly. We conclude that the available body of scientific evidence on the coronavirus disease 2019 holds the key to the development of such strategies and interventions.

Going forward, we recommend that the African research community should scale up research to provide scientific evidence for a better characterization of the epidemiology, transmission dynamics, prevention and control of the virus on the continent.

## Background

The ongoing pandemic of the coronavirus disease 2019 (COVID-19) has severely impacted global health, economy and politics in different patterns in various continents. The disease which is caused by the severe acute respiratory syndrome coronavirus 2 (SARS-CoV-2) was declared a public health event of international concern on 30th January 2020 [1] and a pandemic on 11th March 2020 [2]. As of 10th August 2020, 19 462 112 confirmed cases and 722 285 deaths of the disease had been reported from all countries, areas and territories of the world [3]. Africa reported its first case in Egypt on 14th February 2020 through an importation and by 10th August 2020, all 54 African countries had reported 1 035 932 confirmed cases and 22 920 deaths [4, 5]. Local community transmission has been established in most of these African countries. While Africa remains one of the least affected regions, recent developments and data from within the continent and other regions particularly Europe and America show that the numbers of cases and deaths can grow exponentially and overwhelm even the best of systems in a relatively short time if effective prevention and control measures are not instituted on time [6, 7].

The fear, anxiety and panic induced by the rapid spread of the pandemic prompted several African countries to take drastic actions some of which are not necessarily based on scientific evidence. Some countries closed their air, sea and land borders and imposed total national or partial sub-national lockdowns in a bid to prevent importation and minimize local transmission. Given the weak health system in most African countries, mounting timely and robust responses to the COVID-19 pandemic will be a big challenge hence the need to focus on targeted and high impact prevention and control interventions that could break the chain of transmission quickly. This becomes more pertinent given the African context where inadequate access to water, sanitation and the extended family system renders the implementation of critical preventive measures such as hand washing and social distancing challenging. This is further compounded by the global shortage of required human and material resources, which is more glaring in Africa.

Due to the rapid spread and impact of the disease on human health, trade and travel, several research (mostly preliminary) have been conducted and published to characterize the virus and the dynamics of its transmission, prevention and control. While some of these publications have shown varying findings, conclusions and recommendations, many key and consistent evidences on the characteristics of the virus, its transmission, prevention and control are now emerging. This body of knowledge is critical to inform the development of timely, effective and context-specific prevention and control strategies in Africa. In this article, we review the relevant scientific literatures on the COVID-19 pandemic, and synthesize the relevant evidence that could potentially change the game in Africa’s fight against the disease; finally we propose strategic recommendations for prevention and control of COVID-19 transmission in the Africa continent specifically.

### Evidence-based lessons from literature review

The initial characteristics of COVID-19 cases suggest that the disease is zoonotic [8]. However, recent scientific evidence demonstrates that the current transmission pattern globally is from human-to-human. The virus is similar to the severe acute respiratory syndrome (SARS) and middle east respiratory syndrome (MERS) viruses with susceptibility and severity associated with older age group [9, 10], male gender [11, 12] and underlying medical conditions such as poor immune functions, chronic diseases and surgery [13]. With a basic reproduction rate ranging from 2.6 to 4.71 [14, 15] and fatality rate from 2.3 to 11% [9, 16] it is more transmissible but less fatal compared to SARS and MERS [17, 18]. As is the case with most Influenza viruses, the transmission of SARS-CoV-2 significantly reduces with an increase in temperature and humidity [19, 20], however, this advantage may be offset by the high transmissibility of the virus.

Three main routes of transmission have been identified among humans namely ingestion or inhalation of contaminated droplets released into the air when a patient sneezes, coughs or talks, contact with surfaces which have been contaminated by infected persons and the inhalation of aerosols generated during some medical procedures [21, 22]. A few studies have also suggested faeco-oral transmission of the virus [23, 24]. A recent study concluded that the virus remains viable and infectious in aerosols for a few hours and up to a few days on surfaces, particularly on stainless steel and plastics [25]. While distinct signs and symptoms such as fever, dry cough, runny nose, difficulty in breathing, etc. have been associated with the disease, recent evidence suggests a high proportion of COVID-19 cases are infectious but undocumented either because they have mild or no symptoms but yet continue to transmit the disease [26]. This could contribute to the high transmissibility and rapid geographic spread of the virus.

Based on the lessons learnt from responding to the outbreak in China, prevention and control strategies have been proposed and are in use at various levels. In the absence of a vaccine, infection prevention and control measures that include measures to reduce or prevent exposure to the virus such as identification of suspected cases through syndromic screening at points of entry into countries, public places, health facilities, prevention of shedding of virus into the environment through respiratory hygiene have been recommended as prevention and control strategies in the general population [21]. Others include proper sanitation and waste management, social distancing to prevent contact with infected persons, avoidance of touching potentially contaminated surfaces, eyes, nose and mouth with contaminated hands and hand washing with soap and water or hand sanitizers which contain at least 60% alcohol [21]. Available evidence suggests that social distancing may have a dramatic effect on the transmission of SARS-CoV-2 and other respiratory infections, [27] but there may be renewed virus transmission following relaxation of such measures due to the large proportion of susceptible people that would still be in the population [28]. There is a convergent view on the important role of face masks in reducing the transmission of COVID-19 by protecting healthy persons who come into contact with an infected individual and by preventing infected persons from shedding the droplets into the environment [29, 30]. The use of face masks has also been associated with lower levels of anxiety and depression among the general population and health care workers which further supports its importance in prevention and control of the disease [31–33].

At the individual case level, timely diagnosis, isolation and supportive management of confirmed cases, identification and follow-up of their contacts, prevention of nosocomial transmission through strong infection prevention and control methods and use of personal protective equipment are recommended [34]. There is no known cure for the disease currently but several clinical trials involving various therapies are ongoing. WHO recommends that all laboratory confirmed cases should be isolated and managed in health facility settings but where this is not possible priority should be given to cases with the probability of poor outcomes such as those aged above 60 years and with underlying medical conditions which put them at higher risk [35].

### Recommendations for COVID-19 strategy development specific for the Africa continent

Putting the above scientific evidences on the characteristics and dynamics of COVID-19 transmission, prevention and control into perspective against the backdrop of the social, cultural and economic context in Africa, we deduce several lessons which could guide African countries to better prepare for and respond to the COVID-19 pandemic on the continent.

### Development of tailor-made prevention and control strategies

While the mostly hot and humid African weather and largely younger population may be deterrent factors for wide transmission of the disease, any advantage conferred by this is offset by the high transmissibility of the disease. This is more so given the high population density, larger families and large vulnerable populations such as refugees, Internally Displaced Persons (IDPs), people living with the human immunodeficiency virus (HIV), tuberculosis (TB) and malnutrition thus emphasis in African countries should be on prevention of the spread of infection, especially to these vulnerable groups. In this regard, African countries should invest in identifying, developing and implementing tailor-made prevention strategies to protect at risk populations from infection [36].

### Syndromic surveillance, laboratory testing and contact tracing at the community level

Given the high proportion of undocumented and asymptomatic cases of COVID-19, the use of syndromic surveillance for disease detection at points of entry may not be very effective [37]. While syndromic surveillance may offer some level of reassurance to governments and the general population, the cost in terms of the human and financial resources associated with conducting it may offset its benefits. African countries should rather invest in active search for cases and their contacts at the community and household levels particularly the asymptomatic contacts and transmitters through scaling up testing of all persons who may have been exposed to the virus but remain asymptomatic. To achieve this, COVID-19 testing strategies which prioritize massive testing of various categories of persons based on the transmission scenario should be developed. Additionally, efforts should be made to increase testing capacity and timeliness by decentralizing testing to the sub-national levels.

### Control of the virus at the population level

The evidence that the virus survives much longer on surfaces such as stainless steel and plastic as compared to respiratory droplets have far reaching implications for prevention and control of the virus at the population level. First, risk communication messages should emphasize the high risk constituted by surfaces such as doorknobs, stainless steel handrails, disposable plastics etc. and encourage people to refrain from unnecessary touching of such surfaces. Second, regular disinfection of such surfaces at the household and community level is advised and should be included in risk communication messages and during community engagement sessions [23]. Third, in the light of new findings on asymptomatic transmission of COVID-19 and recommendations on the usage of face masks by healthy individuals, African countries should define clear policies on the use of face masks. Such policies should ensure that face masks are available to those who need them particularly the frontline healthcare workers, caregivers and vulnerable groups, address the issue of shortage of masks and other supplies which is already a challenge in many African countries and importantly provide clear guidance to the general population on the pros and cons, safe use, donning and doffing of face masks. Furthermore, African scientists should pursue urgent researches into the use of locally available material for the production of face masks which are suitable to the African context [29, 30].

### Measures to break the chain of transmission tailored to Africa settings

While available scientific evidence shows that the social distancing which is the ultimate aim of the current lockdowns and population movement restriction measures instituted by several African countries may reduce transmission of the virus in the short term, this strategy alone may not be enough to break the chain of transmission [28]. Since there is already widespread community transmission in many of these countries, population confinement may result in a change in the transmission pattern from the community to the household level [38]. Furthermore, African countries may not be able to sustain such lockdowns for a long time given their socioeconomic context thus they should focus on making the best use of the small window of opportunity that they offer. First, clear objectives should be set for lockdowns which should be to reduce transmission through the scale up of preparedness and response interventions and to control the outbreak in areas of transmission. These objectives should be communicated clearly to the general population to forestall community resistance to lockdowns which is being experienced in some of the African countries. Second, the definition of areas where to impose confinement should be guided by the epidemiology and pattern of transmission of the disease. Third, the lockdowns should be accompanied by intensive risk communication, active case search at the community and household levels, massive testing, contact tracing and isolation. Fourth, adequate preparation should be made to ensure that confined populations have access to basic services such as food, water, healthcare etc. during lockdowns in order to reduce community resistance and ensure adherence. Fifth, appropriate strategies to prevent a second wave of the pandemic following the lifting of the lockdown measures should be developed and implemented [39].

### Use of appropriate case management strategies including home-based care

Given the weak health systems in most of the infected African countries, institutional management of all laboratory confirmed cases may not be a feasible option. On the other hand, home management of such cases is constrained by several challenges due to the large household size, poor housing and high population density in many African countries. Countries should therefore develop context-specific case management strategies which should classify cases according to their risk and health needs, identify places such as health facilities and non-health facilities such as repurposed hostels, schools, hotels or stadia where the various categories of cases will be isolated, define and identify the minimum package of resources such as health workers, medicines, medical equipment and other logistics which are needed to effectively manage the anticipated caseloads. Such strategies should be based on the prevailing transmission scenario in the country.

### Continuation of essential health services and protection of health workers

The COVID-19 pandemic has overwhelmed even strong health systems in Europe and America. A review and analysis of the impact of the 2014–2015 Ebola outbreaks in West Africa on health systems revealed that there was a significant reduction in access to routine health services and this led to substantially increased mortality from preventable diseases such as malaria, measles, HIV, AIDS and TB. African countries should learn from this experience and implement available guidance from WHO to ensure that essential health services are maintained during the COVID-19 pandemic particularly during lockdowns to reduce excess mortality from other preventable diseases [40–42]. Key to maintenance of essential services during the COVID-19 pandemic is the protection of health care workers from acquiring COVID-19 infection; this can be achieved by providing African health workers with the necessary equipment, information and training on how to protect themselves [43].

### Outbreak mitigation in special populations

Management of COVID-19 outbreaks in the situation of population displacement such as refugee and IDPs camps and in prisons and urban slums which are common in Africa is a major challenge. Other high-risk situations include in large-scale industries which employ a large number of semi-skilled workers [44]. The high transmissibility of the virus, overcrowding and inadequate access to social services such as water and sanitation will rapidly facilitate transmission of the virus and constrain implementation of preventive measures such as social distancing in such situations. African countries should, therefore, invest in the development of special public health strategies for prevention and control of outbreaks in such settings [36]. Establishment of COVID-19 information and testing centres near such areas is recommended to improve rapid access of the high-risk populations to COVID-19 prevention and control services [44].

### Mitigation through risk communication for behavioural change

Importantly, the lessons from the rapid spread of the virus in China, Italy, Iran, Republic of Korea and America should be a wake-up call for African countries to rapidly scale up risk communication, community engagement, and participation. The scientific evidence described above and outcomes of anthropological studies on COVID-19 should be used as the basis for development of evidence-based and context specific risk communication messages. Such risk communication messages should be focused on achieving behavioural change and tailor-made to address the several socio-cultural myths, stigma, misconceptions and rumours associated with the virus, its transmission, prevention and control.

## Conclusions

African countries need to act early and decisively to avert excess morbidity and mortality due to COVID-19 and the associated impact it could have on their economy, public health and health system. This could be achieved by using the available global scientific evidence to inform the development and implementation of context-specific COVID-19 prevention and control strategies. Given that there is currently no known cure or vaccine for the disease, such strategies should prioritize prevention and other appropriate interventions in a balanced manner.

The African research community should scale up research to provide scientific information for better characterization of the epidemiology, transmission dynamics, prevention and control of the SARS-CoV-2 and other viruses on the continent. Furthermore, African countries should use the opportunity of the COVID-19 preparedness and response to systematically strengthen their health system capacity for broader and longer-term epidemic preparedness and response by using platforms such as the national action plans for health security. Finally, given the chronic outlook of this pandemic, African countries should explore opportunities to mainstream ongoing COVID-19 response interventions into existing healthcare programmes to ensure cost-effectiveness and sustainability in the long-term.

## Data Availability

Not applicable.

## Abbreviations

AIDS: Acquired immunodeficiency syndrome
COVID-19: Coronavirus disease
HIV: Human immunodeficiency virus
IDPs: Internally Displaced Persons
MERS: Middle East Respiratory Syndrome
SARS: Severe Acute Respiratory Syndrome
SARS-CoV-2: Severe Acute Respiratory Syndrome Coronavirus 2
TB: Tuberculosis
WHO: World Health Organization
